# Paradoxical Tuberculosis Immune Reconstitution Inflammatory Syndrome (TB-IRIS) in HIV Patients with Culture Confirmed Pulmonary Tuberculosis in India and the Potential Role of IL-6 in Prediction

**DOI:** 10.1371/journal.pone.0063541

**Published:** 2013-05-17

**Authors:** Gopalan Narendran, Bruno B. Andrade, Brian O. Porter, Chockalingam Chandrasekhar, Perumal Venkatesan, Pradeep A. Menon, Sudha Subramanian, Selvaraj Anbalagan, Kannabiran P. Bhavani, Sathiyavelu Sekar, Chandrasekaran Padmapriyadarshini, Satagopan Kumar, Narayanan Ravichandran, Krishnaraj Raja, Kesavamurthy Bhanu, Ayyamperumal Mahilmaran, Lakshmanan Sekar, Alan Sher, Irini Sereti, Soumya Swaminathan

**Affiliations:** 1 National Institute for Research in Tuberculosis, Chennai, India; 2 National Institute of Allergy and Infectious Diseases, National Institutes of Health, Bethesda, Maryland, United States of America; 3 Government Hospital of Thoracic Medicine, Tambaram, Chennai, India; 4 Government Rajiv Gandhi Hospital, Chennai, India; Institute of Infectious Diseases and Molecular Medicine, South Africa

## Abstract

**Background:**

The incidence, manifestations, outcome and clinical predictors of paradoxical TB-IRIS in patients with HIV and culture confirmed pulmonary tuberculosis (PTB) in India have not been studied prospectively.

**Methods:**

HIV+ patients with culture confirmed PTB started on anti-tuberculosis therapy (ATT) were followed prospectively after anti-retroviral therapy (ART) initiation. Established criteria for IRIS diagnosis were used including decline in plasma HIV RNA at IRIS event. Pre-ART plasma levels of interleukin (IL)-6 and C-reactive protein (CRP) were measured. Univariate and multivariate logistic regression models were used to evaluate associations between baseline variables and IRIS.

**Results:**

Of 57 patients enrolled, 48 had complete follow up data. Median ATT-ART interval was 28 days (interquartile range, IQR 14–47). IRIS events occurred in 26 patients (54.2%) at a median of 11 days (IQR: 7–16) after ART initiation. Corticosteroids were required for treatment of most IRIS events that resolved within a median of 13 days (IQR: 9–23). Two patients died due to CNS TB-IRIS. Lower CD4^+^ T-cell counts, higher plasma HIV RNA levels, lower CD4/CD8 ratio, lower hemoglobin, shorter ATT to ART interval, extra-pulmonary or miliary TB and higher plasma IL-6 and CRP levels at baseline were associated with paradoxical TB-IRIS in the univariate analysis. Shorter ATT to ART interval, lower hemoglobin and higher IL-6 and CRP levels remained significant in the multivariate analysis.

**Conclusion:**

Paradoxical TB–IRIS frequently complicates HIV-TB therapy in India. IL-6 and CRP may assist in predicting IRIS events and serve as potential targets for immune interventions.

## Introduction

Tuberculosis-associated immune reconstitution inflammatory syndrome (TB-IRIS) is the paradoxical worsening of clinical symptoms, signs and radiological features of TB upon initiation of antiretroviral therapy (ART) after a temporal improvement with anti-tuberculosis therapy (ATT) [1]. This clinical scenario develops despite successful suppression of HIV viremia accompanied by increased CD4^+^ T-cell counts in the blood [1]–[3]. Although such paradoxical reactions have been described in HIV seronegative persons infected with TB [4]–[6], they are far more common in HIV-TB co-infection, with a reported incidence between 8–43% [7], [8]. The clinical manifestations range from fever, tachycardia and minor lymph node enlargement to severe forms manifesting as respiratory distress or neurological deterioration [2]. The pathogenesis of IRIS remains unclear; both antigen-reactive T cells and innate responses have been implicated [9], [10]. The most common predisposing factors for IRIS are pre-ART low CD4^+^ T lymphocyte counts and pre-existing opportunistic infection (OI) or cancer [1]. Treatment of TB before administering ART decreases the risk of IRIS by minimizing the microbial burden at the time of ART-driven immune reconstitution [11]. As findings from large prospective studies and clinical trials have demonstrated that delaying ART can result in increased overall mortality risk associated with AIDS [2], [4], [7], [12]–[14], new guidelines recommend early ART initiation in patients with tuberculosis and severe CD4^+^ T-cell depletion [15]. Thus, it is expected that the IRIS burden will significantly increase in countries with high incidence of TB-HIV co-infection as these guidelines are implemented. Understanding the risk factors and potential biomarkers may help to identify patients at very high risk for IRIS before initiation of ART who might benefit from closer clinical and laboratory monitoring and earlier interventions [16]. Within many other candidate biomarkers, C-reactive protein (CRP) has been systematically described as a very sensitive marker of inflammation and for this reason it has been included in diverse prospective investigations in HIV-infected populations. Several clinical studies have suggested pre-ART levels of CRP can serve as an important biomarker for IRIS and mortality [17], [18]. IL-6 is a cytokine directly linked to CRP and with a critical role in innate immunity against *M. tuberculosis* [19]. Previous studies have also shown increased expression of IL-6 transcripts, secretion in vitro and serum levels in HIV+ individuals developing TB-IRIS by comparison with those not developing IRIS [20]. In addition, IL-6 was one of the cytokines that displayed dramatic reduction in the plasma levels in TB-IRIS patients treated with prednisone [21].

In this prospective study we evaluated the incidence and risk factors of paradoxical TB-IRIS among newly diagnosed culture positive, non–rifampicin resistant pulmonary TB patients with HIV who were ART- naïve and were followed after ART initiation. In addition, we evaluated baseline plasma levels of CRP (an easily accessible laboratory measurement of inflammation) and IL-6 (a cytokine known to induce CRP production) to assess their potential role as predictors of IRIS [18], [20], [22].

## Methods

### Study Design

This TB-IRIS sub-study was nested within a randomized controlled clinical trial (NCT 933790) at the National Institute for Research in Tuberculosis (NIRT), Chennai, India, enrolling HIV patients with newly diagnosed sputum culture confirmed pulmonary TB [23]. The parent randomized trial (ongoing at the time of this report) is comparing daily vs. intermittent ATT regimen in HIV+ patients with pulmonary tuberculosis. The present study was designed to enroll subjects during the first two years of the parent study. Eligible participants for the sub-study had to be above 18 years of age, HIV+ with pulmonary TB confirmed by sputum cultures, harboring rifampicin sensitive *Mycobacterium tuberculosis* at baseline, ART naïve, willing to take drugs under supervision, and willing to sign informed consent. Baseline investigations included 2 rapid HIV tests followed by ELISA, hepatitis B and C serology. Complete blood count (automated hematology analyzer ABX, France), CD4^+^ T-cell count (FACS count flow cytometer, Becton Dickinson, USA), plasma HIV viral load (Roche Amplicor automated viral load monitor, Germany), liver and renal function tests, and random plasma sugar (automated analyzer, Olympus Corporation, Tokyo, Japan) were performed at enrollment. A posteroanterior (PA) chest X-ray (CXR) was performed at baseline and at IRIS event or after 1–2 months of ART in the non-IRIS group per standard clinical practice and was evaluated by two independent experienced readers from a group of five physicians (CC, KS, MA, RK, RR). Three sputum smears were examined by fluorescence microscopy, processed by the modified Petroff’s method and cultured on Lowenstein-Jensen medium (LJ), with species identification and drug susceptibility testing done at baseline and every month until completion of ATT. Positive cultures of *Mycobacterium tuberculosis* were graded as 1+ (20–100 colonies), 2+ (more than 100 discrete colonies) and 3+ (more than 100 colonies forming a confluent mass). For growth up to 19 colonies, exact number of colonies was recorded as previously described [24].

Treatment of pulmonary TB consisted of four drugs: ethambutol (800/1200 mg), rifampicin (450/600 mg), pyrazinamide (1000/1500 mg) and isoniazid (INH) (300/600 mg) for 2 months (intensive phase) followed by INH (300/600 mg) and rifampicin (450/600 mg) for 4 months (continuation phase) given daily or thrice a week, according to the randomization arm in the clinical trial. In regimen 1, drugs were given daily throughout. In regimen 2, the drugs were administered daily in the intensive phase and thrice weekly in the continuation phase, while in regimen 3, ATT was given thrice weekly throughout. All doses were directly observed. ART was started within the intensive phase of ATT, according to Indian national guidelines, after patients were stabilized without overt toxicity to ATT. The ART regimen consisted of either Stavudine (30/40 mg) or Zidovudine (300 mg) given along with Lamivudine (150 mg) twice per day and Efavirenz (600 mg/day) [23]. Patients were hospitalized for ART initiation and were discharged within two weeks. All patients were followed up for 18 months following the start of ATT.

### Ethics Statement

All clinical investigations were conducted according to the principles expressed in the Declaration of Helsinki. Written informed consents were obtained from all participants before enrolling into the sub-study. This study was approved by the Scientific Advisory Committee and Institutional Ethics Committee of Tuberculosis Research Centre, Chennai.

### Diagnosis of IRIS Events

Patients who developed symptoms and signs suggestive of paradoxical IRIS were clinically assessed. CXR and other appropriate investigations were performed if symptoms persisted for at least three days. Blood smear samples were screened for *Plasmodium sp.* and urine cultures were routinely performed to work up febrile episodes. A suspected diagnosis of IRIS was made by a panel of independent physicians (CC, KS, RN, PM, RK) who reviewed the patient’s history, radiographs and physical examination based on case definition guidelines from The International Network for the Study of HIV-associated IRIS (INSHI) [1]. The identified cases of suspected IRIS were adjudicated by a panel of study clinicians (GN, BBA, BOP, IS, SoS) that also required a decline of at least 0.5 log_10_ copies/mL of the plasma HIV viral load with/without an associated increase in CD4^+^ T-cell count for definite IRIS. Enlarged lymph nodes were evaluated with fine needle aspiration or surgical drainage with cultures for *M. tuberculosis* and also for gram positive and negative bacteria. Baseline investigations were repeated at IRIS event in the IRIS group and after 1–2 months of ART in those who were uneventful after ART initiation.

### Plasma Biomarker Measurements

Levels of IL-6 (R&D Systems, Minneapolis, MN), and CRP (eBioscience, San Diego, CA) were assessed in cryopreserved plasma samples maintained at −80°C. The limits of detection were: IL-6, 0.11 pg/mL; CRP, 0.15 mg/L.

### Statistical Analysis

Median values with interquartile ranges (IQR) or frequencies of baseline variables were compared using the Mann-Whitney or Chi-square tests respectively. Multinomial logistic regression was used to evaluate the influence of potential risk factors on the occurrence of IRIS (response variable) including the following independent variables: age, gender, weight, hemoglobin, hematocrit, sputum culture grade, presence of extra-pulmonary or military TB, days to ART initiation, plasma HIV RNA levels, CD4^+^ T-cell count, IL-6 and CRP. For IL-6 and CRP, the relative risk (RR) shown is per standard deviation after log_10_ transformation of each biomarker. The threshold values of IL-6 or CRP plasma levels, which discriminated patients who developed IRIS during the study follow up from those who did not with a high likelihood ratio, were estimated using Receive Operator Characteristics (ROC) curve analyses. The correlation between IL-6 and CRP was assessed using the Spearman test.

## Results

### Baseline Characteristics of the Study Participants

From December of 2009 to November of 2011, a total of 57 HIV positive patients with newly diagnosed culture confirmed PTB (45 males: 12 females), who were ATT and ART naïve, were enrolled in the parent study and 48 of those were included in the TB-IRIS sub-study. Nine patients (18.7%) were excluded due to death prior to ART initiation (n = 3), non-adherence (n = 2), initial culture negative for *M. tuberculosis* (n = 2) and change of ATT before introduction of ART (n = 2). The baseline characteristics of the individuals enrolled in the study are shown in Table 1. Pre-ATT sputum cultures grew *Mycobacterium tuberculosis* susceptible to all first line drugs, except two patients who had pretreatment INH resistance.

**Table 1:** Baseline characteristics of the study participants.

Characteristic	Study population(N = 48)
Male gender	38 (79.2)
Age, median years (IQR)	36 (30–42)
Weight, median Kg (IQR)	41.0 (36.0–47.5)
Time to ART, median days (IQR)	28 (14–47)
***Hematology***	
Hemoglobin, median g/dL (IQR)	9.2 (8.0–10.5)
Hematocrit, median % (IQR)	27.3 (23.3–31.0)
RBC count, median ×10^6^/mL (IQR)	3.5 (3.0–4.1)
CD4^+^ T-cells/µL, median (IQR)	123 (58–187)
CD8^+^ T-cell/µL, median (IQR)	605 (312–958)
CD4/CD8 ratio, median (IQR)	0.18 (0.09–0.35)
HIV RNA, median log_10_ copies/mL plasma (IQR)	5.6 (5.2–5.9)
***Plasma biochemistry***	
ALT, median IU/L (IQR)	30.5 (14.0–48.7)
AST, median IU/L (IQR)	39.0 (25.0–68.5)
***TB evaluation***	
Sputum smear grade	
1+	21 (43.7)
2+	17 (35.5)
3+	10 (20.8)
Sputum culture grade	
1+	9 (18.7)
2+	15 (31.3)
3+	24 (50.0)
Number of chest X-ray zones, median (IQR)	3 (2–5)
Presence of EPTB site	26 (54.2)
Presence of miliary TB	15 (31.3)
ATT regimen*	
Regimen 1	18 (37.5)
Regimen 2	12 (25.0)
Regimen 3	18 (37.5)

**NOTE.** Data represent no. (%) of participants unless otherwise specified. ALT, alanine transaminase; AST, aspartate aminotransferase; ATT, anti-tuberculous treatment; EPTB, extra-pulmonary tuberculosis; IQR, interquartile range; RBC, red blood cell; TB, tuberculosis.

* The ATT regimens differentiated according to the frequency of drug administration (see Methods for full discrimination of the regimens).

### Incidence of IRIS Events

Twenty-six out of 48 patients (54%) experienced IRIS events. The pre-ART characteristics of IRIS vs. non-IRIS are shown in Table 2. The two groups were similar with respect to gender, age, body weight and CD8^+^ T-cell counts and levels of ALT and AST at baseline. Pre-ART hemoglobin was lower in those individuals developing IRIS (median 8.5 vs. 10.1g/dL, P = 0.007), as was the hematocrit (median 25.5 vs. 29.2 P = 0.023). In addition, individuals who experienced IRIS during follow up had significantly lower CD4^+^ T-cell counts than those who did not (median 93 vs. 459, P = 0.005). The median time to ART initiation was 20 days (IQR: 14–30) in IRIS and 43 days (IQR: 23–68) in non-IRIS (P = 0.002). There was no statistically significant difference in the incidence of IRIS between the ATT randomization groups (P = 0.197).

**Table 2:** Baseline characteristics of the study population stratified by IRIS status.

Characteristic	IRIS (N = 26)	Non-IRIS (N = 22)	P-value
Male gender	20 (76.9%)	18 (81.8%)	0.735
Age, median years (IQR)	36 (27–46)	37 (31–40)	0.967
Weight, median Kg (IQR)	42.0 (36.0–48)	40.5 (35.7–46.5)	0.732
Time to ART, median days (IQR)	20 (14–30)	43 (23–68)	<0.001
***Hematology***			
Hemoglobin, median g/dL (IQR)	8.5 (7.1–10.2)	10.1 (9.0–11.3)	0.007
Hematocrit, median % (IQR)	25.5 (20.4–30.6)	29.2 (25.9–33.2)	0.023
RBC count, median ×10^6^/mL (IQR)	3.47 (2.8–3.8)	3.9 (3.2–4.1)	0.087
CD4^+^ T-cells/µL, median (IQR)	93 (39–135)	156 (89–264)	0.005
CD8^+^ T-cell/µL, median (IQR)	765 (311–1095)	459 (297–727)	0.109
CD4/CD8 ratio, median (IQR)	0.09 (0.05–0.18)	0.34 (0.21–0.47)	<0.001
HIV RNA, median log_10_ copies/mL plasma (IQR)	5.9 (5.4–5.9)	5.3 (4.5–5.6)	<0.001
***Plasma biochemistry***			
ALT, median IU/L (IQR)	30.5 (14.0–52.2)	30.5 (13.5–45.7)	0.868
AST, median IU/L (IQR)	54.5 (30.5–91.7)	35.0 (22.7–62.2)	0.102
***TB evaluation***			
Sputum smear grade			0.515
1+	13 (50%)	8 (36.4%)	
2+	9 (34.6%)	8 (36.4%)	
3+	4 (15.4%)	6 (27.2%)	
Sputum culture grade			0.002
1+	6 (23.0%)	3 (13.7%)	
2+	10 (38.5%)	5 (22.7%)	
3+	10 (38.5%)	14 (63.6%)	
Number of chest X-ray zones, median (IQR)	3 (2–5)	3 (2–5)	0.824
Presence of EPTB site	19 (73.1%)	7 (31.8%)	0.004
Presence of miliary TB	12 (46.1%)	3 (13.6%)	0.012

**NOTE.** Data represent no. (%) of participants unless otherwise specified. ALT, alanine transaminase; AST, aspartate aminotransferase; ATT, anti-tuberculous treatment; EPTB, extra-pulmonary tuberculosis; IQR, interquartile range; RBC, red blood cell; TB, tuberculosis.

The IRIS events occurred at a median of 11 days (IQR: 7–16) after ART initiation. Five patients (19.2%) had two episodes and two (7.7%) had three episodes of IRIS. Two patients experienced worsening of symptoms similar to IRIS with ATT itself (prior to ART administration). These patients had recurrence of these symptoms and recrudescence of lesions in the same site in a more pronounced fashion after initiation of ART which were adjudicated as IRIS events (one case of central nervous system [CNS] IRIS and one with pleurisy). The incidence of TB-IRIS in patients with CD4^+^ T-cell counts of <50, 50–99, 100–199 and ≥200 cells/µL was 72%, 63%, 52% and 20% respectively.

**Figure 1:**
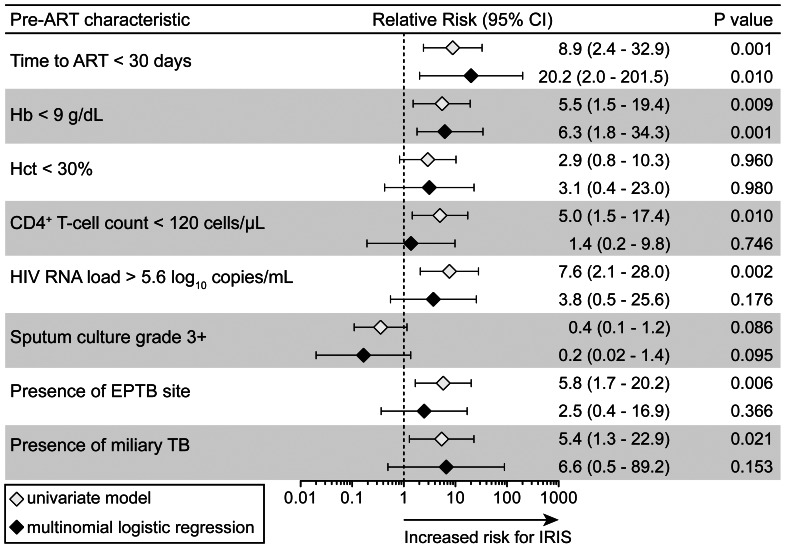
Associations between pre-ART clinical and laboratory characteristics with subsequent TB-IRIS events. Baseline (pre-ART) characteristics significantly different between patients that developed TB-IRIS events in the follow up and those who did not were assessed to test associations with risk for IRIS in univariate and multinomial logistic models. Relative risks (RR) are for values below or above the threshold levels displayed, which were estimated close to median values for the overall study population. Adjustment was performed for all variables presented and also included age and gender. 95% CI, 95% confidence interval.

The overall risk of IRIS within 3 months of ART initiation was associated with a shorter (<30 days) interval between ATT and ART and lower levels of hemoglobin (Figure 1). The unadjusted univariate associations between CD4^+^ T-cell counts, higher HIV RNA levels and the presence of extra-pulmonary or miliary TB at baseline were not significant after adjustment for potential confounding variables (Figure 1).

### Clinical Features and Manifestations of Paradoxical IRIS

Fever with rigor or chills was the predominant presenting symptom of IRIS occurring in all except one patient who presented with generalized tonic clonic seizures and had a brain tuberculoma. None of the patients had *M. tuberculosis* growth in culture at the time of the suspected IRIS event. Worsening lymphadenopathy was the next most common manifestation occurring in 76% (20/26) of patients. Three had Broch’s syndrome (middle lobe collapse consolidation caused by a constriction ring of enlarged right hilar nodes). CNS IRIS was present in four patients, ending fatally in two of them. Radiological deterioration of the CXR was seen in 23/26 patients. Of the 26 patients who experienced IRIS, 15 (57%) had exaggeration, extension or enlargement of pre-existing lesions, two (8%) had appearance of new lesions and nine (35%) had both.

### Plasma IL-6 and CRP as Candidate Biomarkers for TB-IRIS

**Figure 2:**
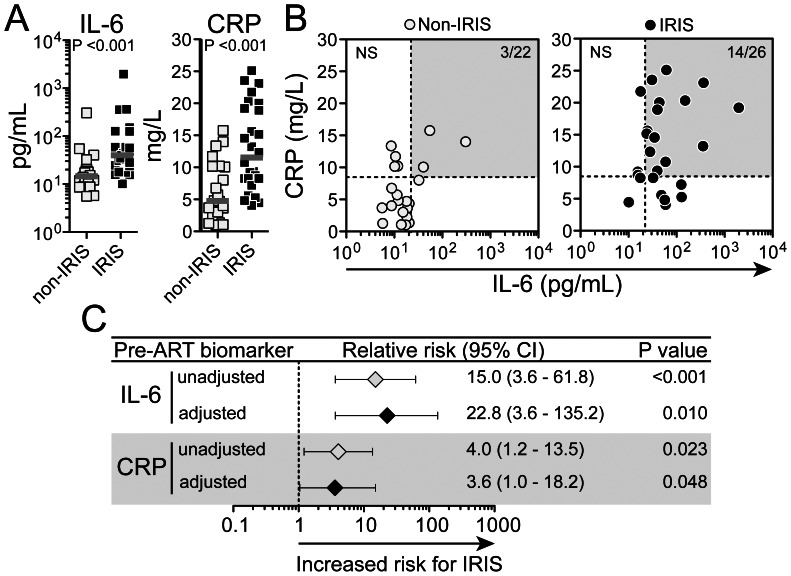
Pre-ART plasma levels of IL-6 or CRP distiguish individuals at higher risk for TB-IRIS. (A) Pre-ART plasma concentrations of IL-6 (left panel) and CRP (right panel) were compared between individuals who developed TB-IRIS during study follow up and those who did not using the Mann-Whitney test. (B) Correlation between IL-6 and CRP values was tested in both groups of patients using the Spearman test. In (B), dotted lines represent the median pre-ART values of IL-6 or CRP in the entire study population. Gray areas highlight those patients displaying values for both biomarkers above their respective median values within the study population. (C) The associations between systemic levels of IL-6 and CRP with risk for subsequent TB-IRIS were assessed by univariate and multivariate models. Relative risks (RR) are per standard deviation increase after log_10_ transformation. RR were adjusted for baseline age, gender, weight, hemoglobin, hematocrit, sputum culture grade, presence of extra pulmonary TB, presence of miliary TB, days to ART initiation, plasma HIV RNA levels and CD4^+^ T-cell count. CI, confidence interval.

Plasma levels of IL-6 and CRP assessed prior to ART initiation were elevated in the patients who experienced IRIS events compared to those who did not (P = 0.015 and P = 0.006, respectively, Figure 2A). The number of persons having levels of IL-6 and CRP simultaneously above the median values of the study population was higher in the group of patients that developed IRIS (14/26, 53.8%) than in those who did not (3/22, 13.6%) (Fisher’s exact test P = 0.023; Figure 2B). CRP and IL-6 displayed similar power to discriminate patients that would experience IRIS from those with uneventful follow up (area under the curves from ROC analysis: IL-6 0.71, P = 0.015; CRP 0.73, P = 0.006); however, the combination of the assays resulted in a sensitivity 12% higher than IL-6 and 16% higher than CRP alone for detection of IRIS (sensitivities: IL-6+CRP = 92%; IL-6 = 80%; CRP = 76%). The univariate associations between IL-6 or CRP and the risk for IRIS persisted after adjustments for age, gender, weight, hemoglobin, hematocrit, sputum culture grade, presence of extra pulmonary or miliary TB, days to ART initiation, plasma HIV RNA levels and CD4^+^ T-cell count (Figure 2C).

### Management and Outcome of IRIS

Patients experiencing IRIS were treated with nonsteroidal anti-inflammatory drugs (NSAIDs) for the first 3–5 days. Prednisolone (0.5–1 mg/Kg/day) was administered for a median of 4 weeks in 21/26 patients who had suboptimal response or persistence of symptoms with NSAIDs. Six patients with more severe manifestations (one with tuberculoma, two with meningitis, and three with significant mediastinal and abdominal adenopathy) required higher doses and longer acting (dexamethasone) or parenteral corticosteroids (methylprednisolone) for 1–2 weeks followed by oral prednisolone at 1–2 mg/Kg of body weight. The median time to resolution or significant improvement of symptoms was 13 days (IQR: 9–23). ART was not interrupted in any patient due to occurrence of IRIS. Two patients died with CNS IRIS (mortality 7.7%). There was one death at month 5 after ART initiation (mortality of 4.5%) in the non-IRIS group, and the cause of death was unclear.

## Discussion

In this study we evaluated prospectively a group of newly diagnosed, sputum culture confirmed, pulmonary TB patients with HIV, who were treatment naïve for both TB and HIV, and followed them after initiation of supervised ATT and ART for emergence of paradoxical TB-IRIS. The diagnosis of IRIS was established taking into account the clinical signs and symptoms including a reduction in HIV viral load, and all cases had AFB culture negativity at the time of the IRIS event. Our findings indicate that more severe immunodeficiency and higher mycobacterial antigen load (extra-pulmonary disease and shorter interval between ATT and ART initiation) along with higher levels of IL-6 and CRP prior to ART initiation are strongly associated with paradoxical TB-IRIS.

The incidence of paradoxical TB-IRIS in this study was high, with over half of the participants developing paradoxical reactions. This could be due to the overall shorter interval between starting ATT to ART initiation, the lower median CD4^+^ T-cell count and the fact that all patients had confirmed (culture-positive) pulmonary TB cases. In addition, closer monitoring and hospitalization for safe initiation of ART made earlier recognition and prompt intervention possible. The closer monitoring and prompt initiation of corticosteroids in cases of IRIS diagnosis might helped an early resolution of signs and symptoms of IRIS, and maybe for this reason the average duration of the IRIS events was shorter than in other studies [4], [11], [16]. Previous studies from the same TB endemic area had shown an incidence of IRIS of 2% among HIV-TB co-infected patients upon initiation of ATT alone, at the pre-ART era [25], and of 21% when ART was started at 8 weeks of ATT [26]. A more recent study from our group investigating patients with CD4^+^ T-cell counts raging up to 63 cells/µL revealed an incidence of 35%, and the IRIS cases were more common in patients with a prior history of TB [27]. In the present study, the median time to ART was only 5 weeks and could have contributed to the higher incidence. Another factor that might have influenced the IRIS incidence was the fact that all the patients had positive sputum cultures for *M. tuberculosis*, whereas in previous studies in the same endemic region approximately 70% of cases were culture confirmed. In addition, there was a higher proportion of extra-pulmonary TB, 52% of the current study participants, contrasting with lower than 25% detected in previous studies [25], [26]. Thus, our findings argue that pre-ART mycobacterial burden is a critical predisposing factor associated with increased risk of IRIS in HIV/TB co-infection.

Several recently published randomized controlled studies including SAPIT, CAMELIA and STRIDE, have shown that ART should be started within the first two weeks of ATT in severely immunocompromised HIV/TB co-infected patients [12]–[14], [28], as this strategy can reduce all-cause mortality despite the higher incidence of paradoxical TB-IRIS. These results do not apply in cases of TB meningitis, as CNS IRIS can be fatal [29]. The most striking results on the impact of early ART initiation on IRIS incidence came from the CAMELIA study, where the hazards ratio of developing IRIS was double in the study arm that had immediate ART initiation than the group of individuals in which ART was delayed, with 10% of the total mortality attributable to IRIS [14]. However, subjects enrolled in the CAMELIA study were sicker and had a median CD4^+^ T-cell count of 25 cells/µL, significantly lower than in our study. In ACTG 5221 study (STRIDE), the median CD4^+^ T-cell count at entry was 77 cells/µL but only 46% of participants had culture-confirmed TB [28]. The incidence of paradoxical TB-IRIS was 11% when ART was started at 2 weeks of ATT compared to 5% when it was started at 8 weeks. In patients with CD4^+^ T cell counts of >50 cells/µL, delaying ART for 8 weeks did not increase risk but showed a definite reduction in IRIS incidence [28]. Consistent with these findings, in our study early ART initiation was a key predisposing factor for TB-IRIS, probably also pointing to a higher antigen burden.

Fevers with rigors or chills were the predominant symptoms of IRIS, along with lymphadenopathy (both cervical and intrathoracic). Previous studies in other populations have shown similar findings [4], [30]–[32] and altogether highlight the importance of lymphadenopathy as a clinical manifestation of paradoxical TB-IRIS. A recent report showed 82% of IRIS events occurring in pre-existing sites and 18% occurring at new sites [33] that are comparable to those reported in our study. The preferential occurrence of IRIS related pathology in sites of ongoing mycobacterial infection reinforces the hypothesis that the immunopathogenesis of this disease involves immune restoration and inflammatory burst in sites overwhelmed by *M. tuberculosis* antigens. Patients were treated with low doses of oral corticosteroids, which were well tolerated and efficacious except in six cases that initially required parenteral steroids. Our results are in agreement with a recent randomized clinical trial in South Africa [16] comparing prednisone and placebo that demonstrated an unequivocal benefit of earlier prednisone administration in cases of suspected IRIS in shortening hospital stay and reducing procedures.

Drug resistant TB stands high in the list of differential diagnoses to consider in cases of persisting clinical deterioration despite ART initiation [4], [34]. Drug resistant TB cases can have an initial improvement on ATT due to the “fall” of drug susceptible strains with subsequent recrudescence of symptoms and signs caused by the “rise” of resistant strains – the so called “fall and rise” phenomenon [35]. Use of corticosteroids in this scenario could potentially have a detrimental effect on prognosis. Hence, ruling out drug resistance, as was done in our study, becomes a critical step before IRIS diagnosis is established, especially when corticosteroid use is contemplated. The introduction of line probe assay in the TB programme in India has helped in diagnosing initial rifampicin resistance [36].

Paradoxical TB-IRIS events can cause significant morbidity and mortality as well as increased expenditure of resources to evaluate and treat patients, making prediction essential for better clinical management. In our cohort, more advanced immunosuppression manifested by lower CD4^+^ T-cell counts, higher HIV RNA levels, and lower CD4/CD8 ratio, in addition to higher mycobacterial antigen load suggested by extra-pulmonary TB foci, lower hemoglobin levels and shorter interval from ATT to ART were all associated with paradoxical TB-IRIS in an univariate statistical model. In multivariable analysis, a shorter interval from ATT to ART and lower hemoglobin levels were independently associated with IRIS. These data suggest that anemia from chronic infection might be an important determinant of IRIS, but this needs to be directly tested. Intriguingly, with the available sample size, there was no association between lower CD4^+^ T-cell counts and higher HIV RNA levels with increased risk of IRIS after adjustment for confounding factors, including time interval from ATT to ART initiation. One possibility is that the ATT alone caused an increase in CD4^+^ T-cell counts pre-ART, and there was interaction between these variables.

The identification of plasma IL-6 and its downstream mediator CRP as potential predictors of TB-IRIS is an important observation considering the need for reliable easily performed biomarkers to facilitate prevention or early interventions. A recent retrospective case-control study in HIV+ patients demonstrated that higher pre-ART plasma levels of IL-6, D-dimer and CRP are associated with increased risk of AIDS events, IRIS or death [18]. Our results confirm that IL-6 and CRP are strongly associated with paradoxical IRIS in the context of HIV/TB co-infection, even after adjustment for other risk factors. Other studies assessing predictors for IRIS in HIV+ patients with co-morbidities other than TB (predominantly *Pneumocystis jirovecii* pneumonia with many patients receiving corticosteroids at ART initiation) found associations with another innate immune cytokine, IL-8, and failed to show involvement of IL-6 [37], arguing that IL-6 might play a more critical or specific role in mycobacterial IRIS. The strong associations between IRIS and markers of inflammation shown here reinforce the hypothesis that activation of myeloid cells by mycobacterial associated molecular patterns [38] may play a critical role in TB-IRIS pathogenesis [3], [10].

The strengths of this study include the confirmation of susceptible *M. tuberculosis* infection by culture positivity, as well as the rigorous microbiologic and clinical follow up including adherence supervision. This allowed us to rule out both bacteriological and virological failure, drug resistance, noncompliance and infection with non-tuberculous mycobacteria at the time of diagnosis of IRIS. The limitations were that the study was nested within a randomized controlled clinical trial with stringent inclusion and exclusion criteria making the pool of candidate study population smaller and possibly introducing a trial referral bias. Nevertheless, the results were overall consistent with previous observations and reinforce the contribution of severe immunosuppression and high antigen load to the onset of IRIS and the potential role of CRP and IL-6 as predictors. Clinical and laboratory predictors of IRIS in co-infected patients starting ART such as those identified here may assist in anticipating complications and facilitate expeditious work up and clinical management.
